# Biofilm formation and prevalence of adhesion genes among *Staphylococcus aureus* isolates from different food sources

**DOI:** 10.1002/mbo3.946

**Published:** 2019-11-25

**Authors:** Qi Chen, Sangma Xie, Xiuqin Lou, Shi Cheng, Xiaodong Liu, Wei Zheng, Zhibei Zheng, Haoqiu Wang

**Affiliations:** ^1^ Hangzhou Center for Disease Control and Prevention Hangzhou China; ^2^ College of Life Information Science and Instrument Engineering Hangzhou Dianzi University Hangzhou China

**Keywords:** adhesion genes, biofilm, polymerase chain reaction, *Staphylococcus aureus*

## Abstract

To assess biofilm formation ability and identify differences in the prevalence of genes involved in biofilm formation among *Staphylococcus aureus* strains isolated from different food samples, the ability of biofilm formation among 97 *S. aureus* strains was evaluated using a colorimetric microtiter plate assay. Thirteen genes encoding microbial surface components recognizing adhesive matrix molecules, and the intracellular adhesion genes were detected by PCR using specific primers. Approximately 72% of the isolates produced biofilms. Among these isolates, 54.64% were weak biofilm producers, while 14.43% and 3.09% produced moderate and strong biofilms, respectively. The *icaADBC*, *clfA/B, cidA,* and *fib* genes were detected in all the *S. aureus* strains, whereas the *bap* gene was not present in any of the strains. The occurrence of other adhesin genes varied greatly between biofilm‐producing and nonbiofilm‐producing strains. However, a significant difference was observed between these two groups with respect to the *fnbpB, cna, ebps,* and *sdrC* genes. No obvious evidence was found to support the link between PFGE strain typing and the capacity for biofilm formation. Considerable variation in biofilm formation ability was observed among *S. aureus* strains isolated from food samples. The prevalence of adhesin‐encoding genes also varied greatly within strains. This study highlights the importance of biofilm formation and the adhesins of *S. aureus* strains in food samples.

## Graphical abstract

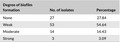

**Highlights**

- A total of 72.16% of *Staphylococcus aureus* isolates from food samples were determined to be biofilm producers.
- The occurrence of adhesion genes varied greatly among *Staphylococcus aureus*.
- A significant difference was observed between biofilm‐producing and nonbiofilm‐producing strains with respect to the *fnbpB, cna, ebps, *and *sdrC *genes.

## 1 INTRODUCTION

*Staphylococcus aureus* is a major foodborne pathogen that causes food poisoning due to the ingestion of heat‐stable staphylococcal enterotoxins (Balaban & Rasooly, 2000; Le Loir, Baron, & Gautier, 2003). *S. aureus* can spread from food handlers, hand contact surfaces, and food contact surfaces during processing and packaging (Sospedra, Manes, & Soriano, 2012). Consequently, *S. aureus* has been repeatedly detected in a variety of foods (Vazquez‐Sanchez, Habimana, & Holck, 2013).

Biofilms are considered a part of the normal life cycle of *S. aureus* in the environment (Otto, 2008), where planktonic cells attach themselves to solid surfaces and subsequently proliferate and accumulate in multilayer cell clusters embedded in special three‐dimensional structures as mushrooms or towers separated by fluid‐filled channels (Azara, Longheu, Sanna, & Tola, 2017). Accumulating evidence indicates that by adopting this lifestyle, bacteria in biofilms gain some advantages over planktonic cells. For example, biofilms can protect this microbe from the action of antibiotic drugs, proteases released by host defense cells and environmental stress factors (Singh, Ray, Das, & Sharma, 2010). This protection may contribute to the persistence of *S. aureus* in food processing environments, consequently increasing cross‐contamination risks and subsequent economic loss due to recalls of contaminated food products (Vazquez‐Sanchez et al., 2013). Therefore, an improved understanding of the development of staphylococcal biofilms at the molecular level is imperative to generate new strategies for biofilm‐associated contamination.

Based on microfluidic flow‐cell systems and time‐lapse microscopy, *S. aureus* biofilm formation has been shown to proceed via a five‐stage developmental process as follows: (a) attachment, (b) multiplication, (c) exodus, (d) maturation, and (e) dispersal (Moormeier, Bose, Horswill, & Bayles, 2014). Accumulating data have indicated the involvement of three factors in these complicated processes, namely eDNA; poly‐β(1,6)‐*N*‐acetyl‐glucosamine (PIA/PNAG), which is induced by coexpression of the intercellular adhesin locus *icaADBC* (Cramton, Gerke, Schnell, Nichols, & Gotz, 1999); and microbial surface components recognizing adhesive matrix molecules (MSCRAMMs), which have been shown to function as extracellular matrix components during early biofilm formation. However, *icaA/B/C* mutants of the UAMS‐1 and USA300JE2 strains demonstrated normal accumulation during the multiplication stage (Moormeier et al., 2014), indicating a major role of these proteins in the development of *ica*‐independent biofilms. *S. aureus* can express a variety of MSCRAMMs, such as fibronectin‐binding proteins A and B (fnbpA/fnbpB) (Cortes, Beltrame, Ramundo, Ferreira, & Figueiredo, 2015; Herman‐Bausier, El‐Kirat‐Chatel, Foster, Geoghegan, & Dufrene, 2015), fibrinogen‐binding protein clumping factors A and B (clfA/clfB) (O'Brien, 2002), biofilm‐associated protein (bap) (Cucarella et al., 2001), serine‐aspartate repeat (Sdr) family proteins (Barbu, Mackenzie, Foster, & Hook, 2014), elastin‐binding protein (Ebps) (Campoccia et al., 2009), collagen‐binding protein (cna), laminin‐binding protein (eno) (Azara et al., 2017), and fibrinogen‐binding protein (fib) (Shannon & Flock, 2004), with the relative impact of each factor appearing to be strain or condition specific (Atshan et al., 2012; Serray et al., 2016; Tang, Chen, Li, Zeng, & Li, 2013).

Therefore, clones isolated from different food sources can differ in their ability to form biofilms. It is unclear whether all MSCRAMMs play important roles in this process. In this study, we investigated biofilm production and evaluated the biofilm‐related genes of *S. aureus* strains isolated from different types of food in markets in Hangzhou.

## 2 MATERIALS AND METHODS

### 2.1 Bacterial strains and growth conditions

Ninety‐seven *S. aureus* isolates from six types of marketed food or associated with food poisoning outbreaks in Hangzhou, Zhejiang Province were investigated (see Table 1). All the isolates were analyzed by cultivation on sheep blood agar and Baird‐Parker agar (Merck) and identified as *S. aureus* by determination of specific properties. *S. aureus* ATCC 6538, which is used as a reference gram‐positive strain in the United States and in European standard bactericidal tests, was used as a strong biofilm‐forming strain in this study. *Staphylococcus epidermidis* ATCC 12228 was used as a negative control based on previous research (Arciola, Baldassarri, & Montanaro, 2001). Bacterial stocks of each strain were maintained at −80°C in tryptic soy broth (TSB) containing 20% glycerol (v/v). All the strains were thawed and subcultured in tryptic soy agar (TSA) for 18–24 hr prior to use.

**Table 1:** Distribution of *Staphylococcus aureus* isolates from different sources

Origin of sample	No. of isolates	Percentage
Food poisoning incidents	39	40.21
Restaurant food	23	23.71
Raw meat	18	18.56
Baked food	10	10.31
Cooked meat product	4	4.12
Fresh juice	3	3.09

### 2.2 Biofilm formation assay

Biofilm formation was measured as previously described by Stepanovic et al. (2007), with minor modifications. Briefly, single colonies from TSA were suspended in 3 ml of TSB and incubated without shaking for 18 hr. The bacterial cultures were adjusted to match the turbidity to that of the 0.5 McFarland standard with phosphate‐buffered saline (PBS). This value corresponds to a cell concentration of approximately 10^8^ cfu/ml for each strain. Then, the cultures were diluted 1:100 in TSB supplemented with 0.5% glucose and added into each well of a sterile 96‐well flat‐bottom microtiter plate (Corning), which was incubated at 37°C for 48 hr under static conditions. After incubation, the planktonic cells were washed five times with sterile PBS, and the adherent bacterial cells in each well were fixed at 60°C for 60 min. Then, the adherent cells were stained with 100 µl of 0.1% crystal violet solution (Sangon Biotech) for 15 min. Excess stain was rinsed off by placing the microplates under running tap water. The plates were then air‐dried, and the stain was dissolved by adding 100 µl of 33% glacial acetic acid (v/v) per well for 10 min at room temperature. The absorbance was read at 490 nm using an iMark microplate absorbance reader (Bio‐Rad Laboratories). The experiment was performed in triplicate at least, and the absorbance of wells containing sterile TSB was used as a negative control. Considering a low cutoff (ODc) to be represented by 3 × *SD* above the mean values of the control wells, the strains were classified into the following categories: no biofilm production (OD ≤ ODc); weak biofilm producer (ODc < OD ≤ 2ODc); moderate biofilm producer (2ODc < OD ≤ 4ODc); and strong biofilm producer (4ODc < OD).

### 2.3 Detection of adhesin genes

Template DNA was obtained from pure cultures of the strains. All strains were grown overnight in TSB. A 1‐ml aliquot of each overnight culture was pelleted by centrifugation at 8,000 × ***g*** for 3 min, resuspended in 200 µl of TE buffer containing 5 µl of lysostaphin (1 mg/ml; Sigma), and incubated for 1 hr at 37°C. The DNA was then extracted using the DNeasy Kit (Qiagen Inc.) according to the manufacturer's recommendations.

The strains were additionally investigated by polymerase chain reaction (PCR)‐based amplification of adhesin genes. The sequences of the primers, the sizes of the PCR products, and the corresponding references are summarized in Table 2. The 25‐µl reaction mixture contained 2.5 µl of 10 × PCR buffer, 1 µl of primers (0.5 µl forward and 0.5 µl reverse), 2 µl of genomic DNA, 1 U of Taq polymerase (TaKaRa Biotechnology Co.), 2 µl of dNTPs (2.5 mM each), and 17.5 µl of distilled water. The mixtures were subjected to the following program in a thermocycler (Bio‐Rad Laboratories): an initial denaturation step at 95°C for 5 min; 35 amplification cycles of 40 s at 95°C, 50 s at different temperatures for different genes and 50 s at 72°C; and an additional extension step of 10 min at 72°C. The PCR amplicons were visualized using UV light after electrophoresis on a 1.5% agarose gel (w/v).

**Table 2:** Primers used in the study

Gene	Nucleotide sequence (5′→3′)	Amplicon size (bp)	Reference
*icaA*	ACACTTGCTGGCGCAGTCAA	188	Rohde, Knobloch, Horstkotte, and Mack (2001)
	TCTGGAACCAACATCCAACA		
*icaD*	ATGGTCAAGCCCAGACAGAG	198	Rohde et al. (2001)
	AGTATTTTCAATGTTTAAAGCAA		
*icaB*	AGAATCGTGAAGTATAGAAAATT	880	Kiem et al. (2004)
	TCTAATCTTTTTCATGGAATCCGT		
*icaC*	ATGGGACGGATTCCATGAAAAAGA	1,066	Kiem et al. (2004)
	TAATAAGCATTAATGTTCAATT		
*icaR*	ATCTAATACGCCTGAGGA	205	Ma et al. (2012)
	TTCTTCCACTGCTCCAA		
*agrI*	ATGCACATGGTGCACATGC	439	Shopsin et al. (2003)
	GTCACAAGTACTATAAGCTGCGAT		
*agr*II	ATGCACATGGTGCACATGC	573	Shopsin et al. (2003)
	TATTACTAATTGAAAAGTGGCCATAGC		
*agr*III	ATGCACATGGTGCACATGC	321	Shopsin et al. (2003)
	GTAATGTAATAGCTTGTATAATAATACCCAG		
*agr*IV	ATGCACATGGTGCACATGC	657	Shopsin et al. (2003)
	CGATAATGCCGTAATACCCG		
*clfA*	ATTGGCGTGGCTTCAGTGCT	288	Tristan et al. (2003)
	CGTTTCTTCCGTAGTTGCATTTG		
*clfB*	CACTTACTTTACCGCTACTTTC	968	Rohde et al. (2001)
	AACGAGCAATACCACTACAACAG		
*fnbpA*	ACCGTCAAACGCAACACAAG	259	O'Neill et al. (2008)
	TTCTGATGCCGTTCTTGGCT		
*fnbpB*	GTAACAGCTAATGGTCGAATTGATACT	523	Tristan et al. (2003)
	CAAGTTCGATAGGAGTACTATGTTC		
*cidA*	AGCGTAATTTCGGAAGCAACATCCA	170	Ma et al. (2012)
	CCCTTAGCCGGCAGTATTGTTGGTC		
*fib*	CTACAACTACAATTGCGTCAACAG	405	Tristan et al. (2003)
	GCTCTTGTAAGACCATTTTCTTCAC		
*bap*	CCATATATCGAAGGTGTAGAATTG	971	Cucarella et al. (2001)
	GCTGTTGAAGTTAATACTGTACCTGC		
*cna*	AAAGCGTTGCCTAGTGGAGA	192	Montanaro, Arciola, Baldassarri, and Borsetti (1999)
	AGTGCCTTCCCAAACCTTTT		
*ebps*	AGAATGCTTTTGCAATGGAT	652	Azara et al. (2017)
	AATATCGCTAATGCACCGAT		
*eno*	TGCCGTAGGTGACGAAGGTGGTT	196	Azara et al. (2017)
	GCACCGTGTTCGCCTTCGAACT		
*sdrC*	ACGACTATTAAACCAAGAAC	560	Azara et al. (2017)
	GTACTTGAAATAAGCGGTTG		
*sdrD*	GGAATAAAGTTGAAGTTTC	500	Azara et al. (2017)
	ACTTTGTCATCAACTGTAAT		
*sdrE*	CAGTAAATGTGTCAAAAGA	767	Azara et al. (2017)
	TTGACTACCAGCTATATC		

### 2.4 Pulsed‐field gel electrophoresis

Pulsed‐field gel electrophoresis (PFGE) of the *S. aureus* isolates was performed in a CHEF Mapper system (Bio‐Rad Laboratories) using chromosomal DNA digested with *Sma*I (New England Biolabs Inc.) according to the conditions described previously by Chung, Jeon, Sung, Kim, and Hong (2008), with some modifications. In this study, DNA from *Salmonella choleraesuis* serotype Branderup H9812 digested with *Xba*I (New England Biolabs Inc.) was included as a molecular size marker. Analysis and interpretation of the banding patterns were performed with Denmark BioNumerics, version 6.6. Pattern similarity was calculated using the Dice coefficient with 1% optimization and a band matching tolerance of 1%.

### 2.5 Statistical analysis

A statistical software package (SPSS 19.0 for Windows; SPSS, Inc.) was used to perform statistical analysis. Differences in gene prevalence between biofilm‐producing groups and nonbiofilm‐producing groups were calculated using the chi‐squared test for each gene. *p*‐values <.05 were considered statistically significant.

## 3 RESULTS

### 3.1 Biofilm formation analysis

A total of 72.16% of the *S. aureus* isolates tested were found to be adherent to varying degrees. Only three isolates (3.09%) were defined as strong biofilm producers; 14.43% of the clones were moderate producers, and more than half (54.64%) were found to be weak producers. A total of 27.84% exhibited no biofilm production (Table 3). The *S. aureus* ATCC 6538 strain was found to be strongly adherent based on the OD490 values, while the *S. epidermidis* ATCC12228 strain was found to be nonadherent based on the OD490 values.

**Table 3:** Analysis of biofilm formation by the microtiter plate method

Degree of biofilm formation	No. of isolates	Percentage
None	27	27.84
Weak	53	54.64
Moderate	14	14.43
Strong	3	3.09

### 3.2 PCR assay

All the primers used in the experiment exhibited specificity with a single band. We detected 13 MSCRAMMs and 5 biofilm‐related genes involved in *S. aureus* cell attachment and multiplication. The results showed that these genes varied among the different *S. aureus* isolates. As shown in Table 4, the *icaA, icaD, icaB, icaC, icaR, clfA, clfB, cidA,* and *fib* genes were all detected (100%), while the *bap* gene was not detected in any of the strains. The prevalence of other related genes among biofilm‐positive clones was as follows: *fnbpA* (+): 100%; *fnbpB* (+): 7.14%; *cna* (+): 78.57%; *ebps* (+): 94.29%; *eno* (+): 97.14%; *sdrC* (+): 94.29%; *sdrD* (+): 77.14%; and *sdrE* (+): 32.86%. In contrast, the prevalence of *fnbpA*, *fnbpB, cna, ebps, eno, sdrC, sdrD,* and *sdrE* in biofilm‐negative isolates was 81.48%, 37.04%, 55.56%, 37.04%, 88.89%, 48.15%, 74.07%, and 33.33%, respectively. As shown in Table 4, significant differences were detected between biofilm‐positive and biofilm‐negative isolates with respect to the *fnbpB, cna, epbs,* and *sdrC* genes. In addition, considering the strain population as a whole, the presence of *sdrC* and *sdrD* significantly improved biofilm formation (Table 5). In particular, the strains with the *sdrC*(+)/*sdrD*(+) genotype exhibited strong or moderate biofilm formation more easily than the other strains. In addition, the *sdrC*(−)/*sdrD*(−)/*sdrE*(−) strains all exhibited no biofilm formation. Isolates that were concomitantly PCR positive for the *sdrC*, *sdrD,* and *sdrE* genes were all positive for biofilm formation. With respect to the *agr* group, *agr*I was the most common type and was detected in 43 (44.33%) of all the isolates. Thirty‐three (34.02%) and 20 (20.62%) isolates were positive for *agr*II and III, respectively, while only one strain was positive for *agr*IV (Table 6). Interestingly, all *agr*III‐positive isolates were able to form biofilms, with isolates 15–80, 15–83, and 16–22 exhibiting the highest biofilm production.

**Table 4:** Correlation between biofilm production and the presence of PIA and MSCRAMM genes

Gene	Biofilm(−)		Biofilm(+)		*p*‐value
	*n*	%	*n*	%	
*icaA*	27	100.0	70	100.0	–
*icaD*	27	100.0	70	100.0	–
*icaB*	27	100.0	70	100.0	–
*icaC*	27	100.0	70	100.0	–
*icaR*	27	100.0	70	100.0	–
*clfA*	27	100.0	70	100.0	–
*clfB*	27	100.0	70	100.0	–
*cidA*	27	100.0	72	100.0	–
*fib*	27	100.0	72	100.0	–
*fnbpA*	22	81.48	70	100.0	–
*fnbpB*	10	37.04	5	7.14	2.63E‐04
*bap*	0	0.00	0	0	–
*cna*	15	55.56	55	78.57	.023
*ebps*	10	37.04	66	94.29	8.48E‐10
*eno*	24	88.89	68	97.14	.099
*sdrC*	13	48.15	66	94.29	1.62E‐7
*sdrD*	20	74.07	54	77.14	.750
*sdrE*	9	33.33	23	32.86	.964

**Table 5:** Relationship of different genotypes and the ability of biofilm formation (BF) in *Staphylococcus aureus* isolates with individual pulsotypes

No. of strains	Pulsotypes	Degree of BF	*sdrC*	*sdrD*	*sdrE*
16–26	C	−	−	−	−
15–22	G1	−	−	+	+
15–23	G2	−	−	+	+
15–56	H1	−	+	+	−
16–18	H2	−	−	+	−
15–45	H8	−	+	+	−
15–46	H8	−	+	+	−
15–47	H8	−	+	+	−
15–54	H8	−	+	+	−
16–16	H9	−	+	+	−
15–57	H10	−	+	+	−
15–58	H11	−	+	+	−
15–43	J1	−	+	+	−
15–60	J1	−	−	+	+
15–63	J1	−	−	+	+
15–85	J1	−	+	+	−
15–55	J2	−	−	−	−
15–61	J3	−	−	+	+
15–62	J3	−	−	+	+
15–64	K5	−	−	−	−
16–20	O	−	−	−	−
15–24	R1	−	−	−	−
15–50	R2	−	+	+	−
15–29	R5	−	+	−	−
15–84	S	−	+	−	+
15–19	V	−	−	+	+
15–18	W	−	−	+	+
15–30	A1	+	+	+	+
15–73	A2	+	+	−	−
15–74	D1	+	+	+	+
15–75	D2	+	+	+	+
16–17	E	+	−	+	−
15–90	F1	+	+	+	−
16–28	F2	+	+	+	−
15–20	H5	+	+	+	+
16–19	H6	+	+	+	−
16−34^a^	H7	+	+	+	−
15–25	I1	+	−	+	−
15–26	I2	+	−	+	−
15–89	J4	+	+	+	+
15–51	K1	+	+	+	−
15–52	K3	+	+	+	−
16–10	K4	+	+	+	−
15–87	L	+	+	+	+
15–96	N	+	+	+	+
15–49	P1	+	+	+	−
15–67	P3	+	+	+	+
15–28	Q1	+	+	−	−
15−31^b^	Q2	+	+	−	−
16–38	Q3	+	+	+	+
16–23	Q4	+	+	+	+
15–41	R6	+	+	+	+
16–21	R7	+	+	−	−
15–48	T	+	+	+	−
16–25	U	+	−	+	−
16–27	X	+	+	−	−
15–86	B	++	+	+	−
15–81	H3	++	+	+	−
15–82	H3	++	+	+	−
16–09	J5	++	+	+	+
16–11	J5	++	+	+	+
16–12	J6	++	+	+	+
16–08	J7	++	+	+	+
16–33	K2	++	+	+	+
16–14	M	++	+	+	+
16–15	M	++	+	+	+
16–49	M	++	+	+	+
15–27	R3	++	+	+	+
15–79	R3	++	+	+	+
16–24	R4	++	+	+	+
15–80	H4	+++	+	+	−
15–83	H4	+++	+	+	−
16–22	P2	+++	+	+	+

a 15 strains isolated from one food poisoning outbreak.

b 11 strains from another food poisoning outbreak.

**Table 6:** The agr types of *Staphylococcus aureus* isolates from this study

	Nonbiofilm‐producing strains *n* (%)	Biofilm‐producing strains *n* (%)	Total *n* (%)
*agr*I	20 (74.07)	23 (32.86)	43 (44.33)
*agr*II	7 (25.93)	26 (37.14)	33 (34.02)
*agr*III	–	20 (28.58)	20 (20.62)
*agr*IV	–	1 (1.43)	1 (1.03)

### 3.3 Determination of genetic relatedness by PFGE

**Figure 1:**
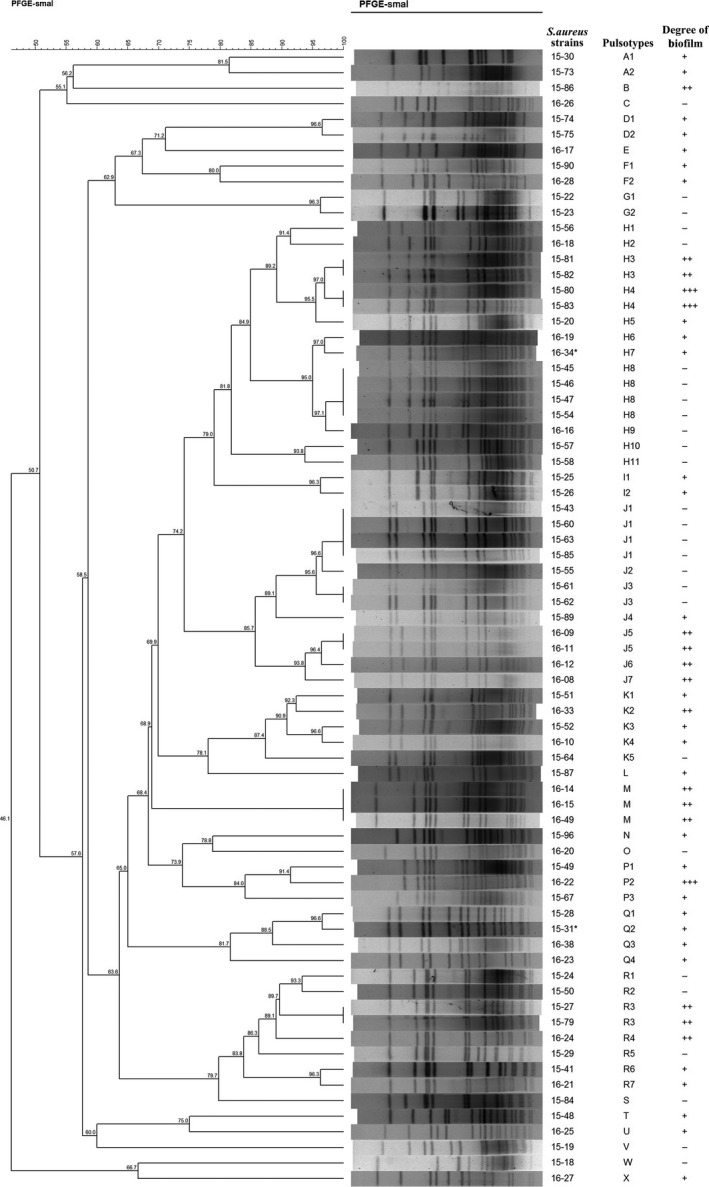
Dendrogram illustrating the percent similarity of PFGE profiles in tested isolates of *Staphylococcus aureus*. *15 strains isolated from one food poisoning outbreak. #11 strains from another food poisoning outbreak

Pulsed‐field gel electrophoresis analysis of *Sma*I‐digested genomic DNA was performed to determine the genetic relatedness of *S. aureus* isolates using the CHEF Mapper system as previously described. Isolates were assigned the same pulsotype if the value of the Dice coefficient of similarity was >80% (Harastani, Araj, & Tokajian, 2014). Clusters were designated with capital letters from A to X (Figure 1). The pulsotype designated with the letter H was the most common, accounting for 21.92% (16/73) of the strains tested, followed by group J, which contained 12 isolates. However, the degree of biofilm formation varied greatly between these two groups.

## 4 DISCUSSION

The ability of *S. aureus* to produce biofilms on surfaces is believed to contribute to food poisoning (Doulgeraki, Di Ciccio, Ianieri, & Nychas, 2017). Biofilm formation involves two crucial steps: primary attachment and accumulation (Azara et al., 2017). An important group of virulence factors that initiate these steps are the MSCRAMM proteins, which are encoded by different genes. Numerous studies have analyzed adhesin genes in *S. aureus* isolates from various sources, including patients with certain infections, animals, airborne particles, food samples, and food poisoning outbreaks (Kouidhi, Zmantar, Hentati, & Bakhrouf, 2010; Nitzsche, Zweifel, & Stephan, 2007; Rahimi, Katouli, & Karimi, 2016; Tang et al., 2013).

In this study, 13 MSCRAMM genes were examined in all 97 *S. aureus* strains isolated from different kinds of food in Hangzhou city*.* Taken together, our results showed that *clfA, clfB, fib,* and *eno* were the most abundant genes among a variety of MSCRAMM genes, with percentages ranging from 75% to 100%. No difference was observed in the prevalence rate of the above genes among biofilm‐negative and biofilm‐positive strains. However, Emaneini et al. found that a highly significant difference was present in the prevalence of some MSCRAMM genes, such as *ebps, eno, fnbpA/B,* and *fib,* among these two groups. This observation is partly consistent with other studies that showed the percentage of *fnbpA* and *clfA* in biofilm‐producing strains to be significantly higher than that in nonbiofilm‐producing strains (Rahimi et al., 2016).

The fibronectin‐binding proteins of *S. aureus* are important virulence factors and contribute to bacterial adhesion (Shanks et al., 2008). For *S. aureus*, *fnbpA* and *fnbpB* have been described, and these genes share a high degree of sequence similarity (Jonsson, Signas, Muller, & Lindberg, 1991). Based on the results that mutation of *fnbpA* or *fnbpB* alone did not affect biofilm formation and expression of either gene alone from a complementation plasmid in *fnbpA* and *fnbpB* double mutants could restore biofilm formation, O'Neill (2008) indicated that the roles of FnbpA and FnbpB in biofilm formation were complementary. In the present work, *fnbpA* was detected in almost all the isolates, and no obvious difference was observed regardless of biofilm production, which is consistent with the results of previous studies (Atshan et al., 2012; Pereyra et al., 2016; Tang et al., 2013). However, for *fnbpB*, the results were complicated. In our study, the occurrence of the *fnbpB* gene was low, especially in the biofilm‐producing group (7.14%). This low detection rate of *fnbpB* was also observed in the studies conducted by Emaneini, Khoramrooz, Shahsavan, Dabiri, and Jabalameli (2015), Atshan et al. (2012), and Vergara et al. (2017), while Tang et al. (2013), Pereyra et al. (2016), Filipello et al. (2019) observed a high prevalence of this gene (68.75%, 90%, and 73.6%, respectively). Furthermore, all moderate and strong biofilm‐forming isolates in our study (designated as ++ or +++) were shown to be *fnbpB*‐negative. This result was similar to that of the study conducted by Filipello et al. (2019), where the *fnbpB* gene was not detected in two isolates with strong biofilm‐forming ability. As in many reports, the *bap* gene was not detected in any isolate (Khoramian, Jabalameli, Niasari‐Naslaji, Taherikalani, & Emaneini, 2015; Tang et al., 2013; Vautor, Abadie, Pont, & Thiery, 2008; Vazquez‐Sanchez et al., 2013). The *bap* gene is present in the pathogenicity island SPIbov2, which has been identified in only a small proportion of *S. aureus* isolates (Vautor et al., 2008), originating only from bovine subclinical mastitis (Cucarella et al., 2001), even though bap was the first protein reported to be involved in *S. aureus* biofilm formation. The gene *cna* is the only recognized *S. aureus* gene that encodes an adhesin that specifically binds to collagen (Patti et al., 1995). Our results showed that *cna* was present significantly more frequently in biofilm‐producing strains than in nonbiofilm‐producing strains. Another study reported that *cna*‐positive isolates (20%) were identified as moderate or strong biofilm producers (Pereyra et al., 2016). In contrast, Khoramian et al. (2015) found that there was no obvious difference in the prevalence of the *cna* gene between these two groups, which is consistent with the findings of Tang et al.

The elastin‐binding protein of *S. aureus* (ebps) is an adhesin that is responsible for attachment to host cells via binding to elastin (Park, Rosenbloom, Abrams, Rosenbloom, & Mecham, 1996). However, inactivation of ebps has a minimal effect on the binding of *S. aureus* to immobilized elastin (Roche et al., 2004). The *ebps*‐deficient strain not only continued to form biofilms but also exhibited significantly enhanced biofilm formation at high concentrations of Zn^2+^, suggesting that ebps can regulate biofilm formation by binding not to proteins but to Zn^2+^ (Nakakido, Aikawa, Nakagawa, & Tsumoto, 2014). In this study, 66 of 72 (94.29%) biofilm‐positive *S. aureus* isolates were PCR positive for the *ebps* gene, while the percentage of biofilm‐negative strains was only 37.04%. However, Azara and his coworkers showed that 80.6% of the *S. aureus* isolates collected from ovine mastitis samples possessed the *ebps* gene. Another study demonstrated that *ebps* was detected in all MSSA and MRSA clones regardless of the capacity for adhesion (Atshan et al., 2012). Taken together with previous conclusions, these results show that further research may be needed to elucidate the role of the *ebps* gene in the biofilm formation process.

A recent study compared the biofilm development of the parent strain *S. aureus* Newman (a strain that does not express SasG and does not anchor fnbps on the cell wall) with that of *sdrC*, *sdrD,* and *sdrCDE* mutants (Barbu et al., 2014). The *sdrC* mutant exhibited significantly inhibited biofilm formation, whereas the *sdrD* single mutant was not affected. The *sdrCDE* knockout strain exhibited decreased capacity of biofilm formation compared to the wild‐type strain, which was consistent with our results that isolates that tested negative for the *sdrC*, *sdrD,* and *sdrE* genes by PCR were all negative for biofilm formation. However, complementation of strains defective in the cell wall‐anchored (CWA) proteins clfA, clfB, IsdA, IsdB, sdrC, sdrD, and sdrE with sdrC restored biofilm formation. Another study that investigated the expression of different genes in clinical isolates from skin demonstrated that the *ica* operon and *sdrC* are highly expressed in response to biofilm formation (Shin et al., 2013). Based on our result that 94.29% of biofilm‐positive isolates carried the *sdrC* gene, *sdrC* may be an important molecule for bacterial intercellular binding and subsequent biofilm formation.

The *agr* sensing system has been shown to downregulate genes of cell wall‐associated adherence factors, leading to decreased biofilm initiation (Moormeier & Bayles, 2017). In this study, *agr*I was the dominant *agr* type among the tested *S. aureus* strains (44.33%), followed by *agr*II (34.02%) and *agr*III (20.62%), which was consistent with the results of previous studies (Bardiau, Detilleux, Farnir, Mainil, & Ote, 2014; Bar‐Gal et al., 2015; Filipello et al., 2019; Khoramrooz et al., 2016; Mitra et al., 2013). Furthermore, it was interesting that all of the *agr*III isolates were identified as being biofilm positive, 11 of which were strong/moderate biofilm‐producing strains. This result was also similar to those of the studies conducted by Khoramrooz et al. (2016) and Rahimi et al. (2016). Thus, it is likely that there is a significant association between *agr*III and biofilm production in *S. aureus* isolates.

A second group of virulence factors that contribute to biofilm formation is PIA/PNAG, which is synthesized by *icaADBC* operon‐encoded enzymes (O'Gara, 2007). In this study, all isolates tested were found to be positive for the *icaADBC* genes. These findings were similar to the observations of Atshan et al. (2012) and Arciola et al. (2001), as there was no difference in the distribution of the *icaA* and *icaD* genes in the biofilm‐positive and biofilm‐negative strains. However, the prevalence rates of the *icaA* and *icaD* genes vary greatly among different studies. For example, when *S. aureus* is exposed to different temperatures and contact surfaces for different amounts of time, distinct gene expression profiles can be observed (Atshan et al., 2013; Kroning et al., 2016; Stanley & Lazazzera, 2004). This finding is an indication of high variability and, at least based on biofilm mass production, suggests that the presence of genes encoding PIA/PNAG is not an absolute determinant of biofilm formation ability.

Pulsed‐field gel electrophoresis has been considered a gold standard for typing *S. aureus* strains due to the high discriminatory power and reproducibility of this technique. There is little evidence to support the link between PFGE strain typing and the capacity for biofilm formation. Smith et al. (2008) divided 763 MRSA isolates from hospitals throughout Scotland into three main clonal types (EMRSA‐15, EMRSA‐16, and sporadic isolates) based on PFGE genotyping results and demonstrated that EMRSA‐15 isolates formed a significantly greater number of moderately and fully established biofilms than EMRSA‐16 isolates. Similar to other studies (Naicker, Karayem, Hoek, Harvey, & Wasserman, 2016; Pereyra et al., 2016), the number of tested isolates in the present work was too small to analyze biofilm formation based on PFGE clusters due to the high variability in the results. Multilocus sequence typing (MLST) and spa lineages may serve as genetic predictors of biofilm formation. Further studies are required for MLST or spa typing of these clones.

## 5 CONCLUSIONS

In general, considerable variation in biofilm formation ability was observed among *S. aureus* strains isolated from food samples. The prevalence of adhesin‐encoding genes also varied greatly within strains. There was no significant difference in the prevalence rate of MSCRAMM genes among the nonbiofilm‐producing isolates and among those producing weak, moderate, and strong biofilms, except for *fnbpB*,* cna*,* ebps*, and *sdrC*. Our results, in combination with those of previous studies, indicate that detection of *sdrC* is a practical approach for the prediction of biofilm formation. Further research on a large number of isolates may be needed to verify this possibility and explore the connection between the genetic background of *S. aureus* and the biofilm formation ability based on microbial subtyping.

## CONFLICT OF INTERESTS

None declared.

## AUTHOR CONTRIBUTIONS

QC designed and performed all of the experiments described above and wrote the paper. SMX analyzed the results. XQL helped with the conceiving of the study and the manuscript draft. SC and XDL performed the experiments after first revision. HQW, WZ, and ZBZ purchased materials and participated in the study's coordination.

## ETHICAL STATEMENT

None required.

## Data Availability

All data associated with the article have been included in this manuscript.
